# Muscular Damping Distribution Strategy for Bio-Inspired, Soft Motion Control at Variable Precision †

**DOI:** 10.3390/s23052428

**Published:** 2023-02-22

**Authors:** Patrick Vonwirth, Karsten Berns

**Affiliations:** Department of Computer Science, RPTU Kaiserslautern-Landau, 67663 Kaiserslautern, Germany

**Keywords:** bio-inspired, impedance, control, artificial muscles, series elastic actuator, humanoid

## Abstract

Bio-inspired and compliant control approaches have been studied by roboticists for decades to achieve more natural robot motion. Independent of this, medical and biological researchers have discovered a wide variety of muscular properties and higher-level motion characteristics. Although both disciplines strive to better understand natural motion and muscle coordination, they have yet to meet. This work introduces a novel robotic control strategy that bridges the gap between these distinct areas. By applying biological characteristics to electrical series elastic actuators, we developed a simple yet efficient distributed damping control strategy. The presented control covers the entire robotic drive train, from abstract whole-body commands to the applied current. The functionality of this control is biologically motivated, theoretically discussed, and finally evaluated through experiments on the bipedal robot Carl. Together, these results demonstrate that the proposed strategy fulfills all requirements that are necessary to continue developing more complex robotic tasks based on this novel muscular control philosophy.

## 1. Introduction

Artificial cognition capabilities grow with the development of better sensors, and computer-guided decision capabilities grow with the availability of computational power. Equivalently, robotic locomotion is changing with the continuous development and improvement of actuation systems. The available drive technology is highly specialized and tailored to the exact needs of the surrounding machines. Different types of wheels have been developed to drive over different terrains. Today, stiff motors with large gear ratios are used to precisely control industrial robot arms on specific trajectories. Legged locomotion, however, requires different properties of an actuation system than most industrial applications. Compared to other tasks, the biggest problem of legged locomotion is likely the physical impact of footsteps, which can damage stiff mechanical systems. Therefore, control strategies for legged locomotion should differ from the well-studied approaches implemented in today’s stiff and inflexible industrial automation robots.

In order to develop such a different motion strategy, this paper distances itself from classical control theory. Instead, a natural, musculoskeletal locomotor system was used as a blueprint to develop the new motion control philosophy. The introduced concept covers all involved elements of a robotic drive system, including the low-level motor control as well as the abstract, high-level input. To the best of our knowledge, no other approach exists that tackles the problem of natural, compliant motion control in an equivalent manner. The article at hand, first published in the proceedings of the 25th International Conference on Climbing and Walking Robots (CLAWAR 2022), is an extended description of the concept introduced in [1]. Special focus is given to the distributed muscular control details.

Within the following, an introduction to the variety of existing robotic actuation systems, including control approaches, is given. We focus on the bipedal robot Carl in Section 1.2. The development description of the new, compliant control begins in Section 2 at the muscular level. With the coverage of higher abstraction levels, Section 3 finalizes this description. Section 4 contains a theoretical, in-depth analysis of the capabilities and limits of the proposed strategy. An experimental evaluation and discussion are given in Section 5. Finally, Section 6 concludes the findings and offers an outlook on further developments that can be based on the proposed, novel control strategy.

### 1.1. Drive Technology for Artificial Legged Locomotion

In the past decades, many different approaches have been developed to make robots walk. Aside from classic concepts, such as the so-called *divergent component of motion* [2,3,4,5] or *hybrid zero dynamics* [6,7,8,9], natural, bio-inspired concepts, such as muscle-reflex strategies [10,11,12], machine learning approaches [13,14], or the *B4LC* (Bio-inspired Behavior Based Bipedal Locomotion Control) [15,16,17] system were developed and evaluated in simulations and on real robotic systems. Despite the fact that these strategies are opening up a wide variety of approach basics, all of them share one property—all approaches are developed upon (and, hence, rely on) a concrete actuation system, e.g., position or torque controllability, or stiff or compliant mechanical structures. Locomotion strategies advance with the progress of motor technology and cannot be separated from the controlled hardware. Therefore, the new control philosophy introduced in this article also includes a novel physical actuation concept derived from natural muscle properties.

Unfortunately, due to the past weaknesses of actuation systems (w.r.t. the needs of legged locomotion), the actual hardware capabilities have strongly limited the research possibilities. As a result, legged locomotion only played a minor role in past mobile robotic research. With the invention of soft actuation systems, however, this area of research is about to receive more interest. Research on legged locomotion has progressed in the context of quadruped robots, including research on *MIT Cheetah* [18,19], *HyQ* [20,21], *ANYmal* [22,23], and *Spot* [24].

Ficht and Behnke [25], within their short overview on the technological and development process of humanoid robots, clearly show the recent trend toward so-called *soft robotics*. Obviously, this directly addresses the above-mentioned problem with external impacts. In most cases, the softness of a robot is achieved by using so-called *artificial muscles*. The most popular types of artificial muscles used for locomotion are pneumatic, hydraulic, and electric drives, or a combination of these. They all come with physically integrated, serially attached, or high-speed controlled elasticity and/or transparency. A quite unorthodox example of a pneumatic drive system is a fully inflatable upper-body humanoid introduced by Best et al. [26]. The hydraulic, so-called *integrated smart actuator* introduced by Barasuol et al. [27] is one of the latest developments in hydraulic drive trains that is meant to be integrated into the next generation of the quadruped robot *HyQ*. Electrically powered artificial muscles are, in most cases, *quasi-direct drives* [25]. This architecture considers the required force, velocity, and actuator transparency in accordance with the needs and physical limits. A rotational example of such an actuator, introduced by Seok et al. [28], has been built into the *MIT Cheetah* robot. In contrast, the *compliant robotic leg* Carl [29,30] (which is used as an evaluation platform throughout this article) is equipped solely with linear *series electric actuators* (Seas), the so-called RRLab Sea*s* [31]. A hybrid electric–pneumatic actuation concept was proposed by Mohseni et al. in [32]. This actuation concept is also used to better reproduce natural leg construction and control characteristics.

Moreover, artificial muscles include a large variety of large and small-scale technologies suitable for all types of motion, e.g., grasping. A recent overview of such technologies is given by Mirvakili and Hunter in [33]. However, in comparison to humans and animals, it is clear that there is still a lot of room to improve current technical systems and to learn from nature.

### 1.2. Bio-Inspired, Muscular Actuation of Carl

**Figure 1:**
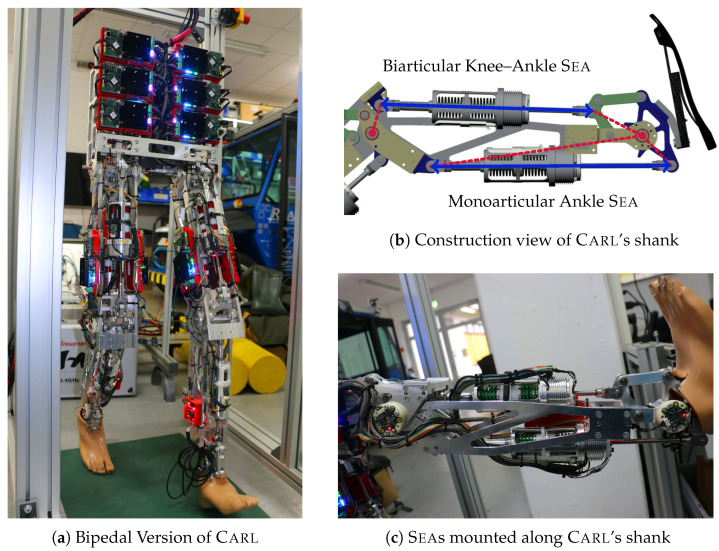
The bipedal version of the robotic leg Carl consists of a short trunk and two planar, muscular-driven legs with medical standard prosthetic feet.

The bio-inspired locomotion strategy *B4LC*, developed by Luksch [16] in 2010, is proven to be principally functional in a pure simulative environment. The basic idea of this concept is to combine simple, torque-based feed-forward control patterns with reactive, impedance-based reflexes. By extending this approach with more advanced skills, such as upslope walking [17], walking on rough terrain [34], and stepping over obstacles [35], the basic idea of the *B4LC* approach seemed to be suitable for real-world evaluation. Due to the high demand on the actuation system, the first successful attempt to construct and transfer this pure virtual control concept to a planar robotic leg was achieved by Schütz in 2020 [36]. Equal to the natural motorization principles, the planar, bipedal robot Carl (Figure 1) [29] is equipped with both, mono- and biarticular electrical Seas (Figure 1b,c) [31].

Stand-alone experiments on the bipedal version of Carl, although successfully demonstrated as a single leg being mounted over a treadmill, have failed for two main reasons. (1) The applied feed-forward torque patterns should offer basic motion characteristics. Due to the changing environmental conditions (e.g., temperature and dust-dependent friction), noisy measurements, and imprecise motor actions, these patterns had to be adapted permanently while creating a large variety of resulting motions. (2) There are too many nested and mechanically coupled control loops. The unmodeled and unconsidered interactions of all individually controlled Seas artificially lowered the stability of the overall system far below its theoretical capabilities and far below the requirements of free walking.

Hence, with a special focus on these two issues, a new low-level, muscular control strategy for compliant and imprecise locomotion is described as follows: It is a strongly simplified, bio-inspired concept of distributed damping control based on series elastic actuators that imitate muscles. In contrast to other state-of-the-art approaches, classical control goals, such as high tracking precision, are explicitly not desired. For most natural motions, such as walking, it is absolutely sufficient to *roughly* track a desired trajectory, while the ability to react and adapt to the environment is of higher interest.

## 2. Actuation Concept for Compliant Control

From the need to be impact-resistant, the requirement directly evolves to include mechanical, compliant elements in the construction of a robotic leg. Unfortunately, every element adds a passive, uncontrolled degree of freedom to the robot. Hence, during the mechanical design phase of a robotic limb, impact tolerance and controllability have to be balanced against each other.

### 2.1. Analysis of Impedance Control Strategies

In 2015, Semini et al. [21] proposed following the principle of *stiff inside, soft outside*, a guideline for designing impact-tolerant robotic limbs. This principle suggests building the actuated limb as stiff as possible in order to allow for classic, high-precision control. Environmental impacts are compensated for by compliant contact points, e.g., spring-loaded or rubber feet. The most significant advantage of this approach is its proximity to the traditional, stiff construction design. By definition, it offers the possibility to apply well-known, standard trajectory control algorithms on such a robot design. Hence, it is quite common in legged robotics and has been successfully demonstrated on several robots, e.g., on the bipedal robot *DURUS* [37,38] and the quadruped robots mentioned in Section 1.1. However, the advantage of having only a single compliant element close to the end of a limb also forms the biggest drawback of this approach. The robot is limited to interacting with the environment only through the specially designed contact points and in no other way than what these elements are designed for.

To overcome this limitation of interaction, a whole-limb impedance control has to be provided. It can be realized by mechanical compliance, virtual compliance, or a combination of both. Mechanical compliance offers impact tolerance at the cost of controllability whereas virtual compliance does the opposite. In [21], Semini et al. focused on controllability and suggested using pure active impedance control since *“no springs are needed to protect the actuation system”*. However, their principle of *stiff inside, soft outside* can be interpreted as already violating their above-mentioned statement.

Approaching the problem of impedance control from the mechanical side can be done by using soft Seas, such as the so-called RRLab*-*Sea, introduced and successfully evaluated by Schütz et al. [31,39,40]. However, although successfully tested as a single, isolated actuator, the combined control of five of them mounted in the robotic leg Carl uncovered serious stability issues. The nested and mechanically coupled control loops form a critically large number of unhandled oscillation circuits inside the robot. From low-level to high-level, the mechanical and digital oscillating systems are as follows:

- The electric motor’s mass against the serial spring;
- The unconventionally by-passed PID spring-deflection controller;
- The outer-loop impedance controller of the whole actuator surrounding the inner-loop force controller;
- The mechanical and virtual springs of several Seas that are connected to one shared joint;
- The high-level force distribution strategy of all available actuators;
- The physical coupling of both legs on the bipedal version of Carl.

Details about the realization of the mentioned RRLab-Sea controller are given in [36]. Information about the tested force distribution strategies can be found in [30,41]. To keep the overall system stable, the capabilities of all Seas had to be limited far below the theoretical requirements needed to execute stable locomotion with Carl.

From these points of view, it seems better to compromise between physical and virtual impedance.

### 2.2. Natural Muscle Emulation on a Series Elastic Actuator

A natural muscle can be described as a composition of two main parts, i.e., a motor part, which is the contractile element, and an elastic element, which is the serially attached tendon. In between both, the so-called *Golgi tendon organ* is located, sensing changes in the muscle tendon tension. This organ is used to control and protect muscular tension, e.g., the commonly known muscle stretch reflex is triggered by that organ [42].

From the mechanical point of view, a Sea is a nearly perfect technical platform to emulate muscular behavior. Using an electrical Sea, the only disadvantage compared to a biological muscle is the reflected inertia of the rotating electric motor. However, the above Section 2.1 clearly points out the big problems that arise from the limited possibility of stably controlling such a Sea to mimic an ideal impedance. As the only consequent result, we propose a trade-off of the introduced pure virtual impedance control (i.e., following the principle of *stiff inside, soft outside*) against the usage of series elastic actuation. That is to shift the principle of *stiff inside, soft outside* from the abstract limb level into the limb itself to the actuator level, i.e., to use the mechanical structure of the Sea but without trying to control its elasticity. By doing so, most of the previously described instability causes can be avoided by design. In fact, they are simply removed from the design of the controller. Obviously, this comes at the cost of control precision. The need for high-precision force sensing vanishes, and the elastic element, i.e., the *soft-outside* part degenerates to only protect the stiff, internal drive train from physical impacts. As a direct result, much higher stiffnesses can be used, extending the range of controllable frequencies. However, due to the lack of feedback and control of the elastic element, impacts have to be handled by the actuator’s transparency, i.e., its backdrivability, which becomes a mandatory property.

In the mid-1980s, Mussa-Ivaldi et al. [43] found that the short-range end-point stiffness of a human arm features three important properties:

- Measured stiffness matrices at the end-point of a human arm can be treated as symmetric, i.e., elliptical;
- The general shape/orientation of the end-point stiffness ellipse mainly depends on the arm configuration, not the muscle activities;
- Muscular co-contraction strongly increases the overall size of the measured stiffness ellipse, i.e., its spring rate.

**Figure 2:**
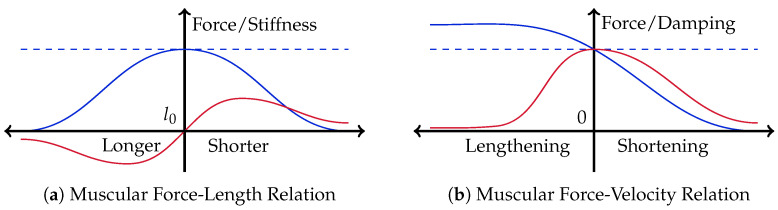
Sketches of the two principal impedance properties of a natural muscle. On the left, the (**a**) stiffness characteristic is shown, on the right, the (**b**) damping. The overall emitted forces are represented by the blue curves. The dashed line indicates the maximum force at rest. In red, the derived shapes of the muscle-intrinsic stiffness and damping values are sketched.

These insights support the assumption that the (short-range) end-point stiffness is generated on a muscular level, exploiting the physically inherent dynamics of the contractile elements, tendons, and mounting positions of the muscles. Recent studies on the effects of having muscle-like dynamics built inside actuators support this hypothesis. The pure existence of muscle-like force characteristics significantly reduces the amount of control information required from some central intelligence [44]. The general types and shapes of these characteristics are well known and create a *muscle-level, zero-delay feedback system*, the so-called *preflexes* [45,46]. There are three main dependencies: time, length, and motion. While the temporal force dependency (i.e., muscular fatigue) can be ignored as an unnecessary limitation, the two others construct the two impedance properties, i.e., *stiffness* and *damping*, respectively. In general, the emitted force of a muscle with respect to these two separated impedance properties can be estimated as follows:

(1) $$\begin{array}{ccc} F\left(l\right)∼F_{Max}-K\left(l\right)\;l & \; & F\left(v\right)∼F_{Max}-D\left(v\right)\;v \end{array}$$

with $F_{Max}$ being the maximum muscle force at rest and K(·) and D(·) representing stiffness and damping, respectively. Both impedance functions are modeled in such a way that the individually resulting overall emitting forces meet their known shapes, given in [46]. Figure 2 sketches these two properties together, interpreted as intrinsic impedance.

It can be seen that muscular stiffness contains both positive and negative sections. The muscular stiffness is partially unstable and not really suitable to be taken as additional muscular control property. Furthermore, stiffness requires an additional equilibrium position as a secondary control. Damping, on the other hand, stays positive, i.e., stable, over the whole range of motion. From this technical point of view, the muscular *preflexes* should be reduced to only cover muscle-intrinsic damping. That reduction is also supported by Brown and Loeb in their publication about *preflexes* [45]. They explicitly observed the force-length relation of a muscle to be, more or less, irrelevant compared to the effect of the force–velocity relation.

Muscular damping can be viewed as a decentralized stabilization mechanism of the natural muscular actuation system. Additionally, unlike force, damping is a non-directional property. Forces can be offset by antagonistic muscles, whereas antagonistic damping always adds up. Therefore, muscular co-contraction can be employed to increase damping, irrespective of the force that serves as a lower bound for damping.

With the increment of damping of the contractile element of a muscle, the stiffness of its attached tendon also increases to dominate the overall muscular behavior. While zero damping results in no spring-like behavior at all, infinite damping, i.e., no motion of the contractile element, leads to the muscle becoming its tendon. The observable end-point stiffness of a limb can be stiffened using muscular co-contraction while its general shape mostly stays related to the limb posture as already mentioned above [43]. Muscular co-contraction, i.e., damping, can be used to stiffen up a limb, to increase tracking precision and resistance to external disturbances. Unfortunately, increasing damping comes at the cost of higher energy consumption. Various studies on disturbed natural motions support this theory by observing a rise of muscular co-contraction to counteract unexpected disturbances or to increase accuracy [47,48,49].

With the introduction of damping as the only secondary control property next to force, an overall consistent concept of artificial, muscular control of a series elastic actuator is defined. One Sea represents a pair of two antagonistic muscles. The two independent muscular activations are represented by the two individual controls offered by a single Sea: force and damping.

(2) $$\begin{array}{ccc} F\left(a_{1},a_{2}\right)∼a_{1}\;F_{Max}-a_{2}\;F_{Max} & \; & D\left(a_{1},a_{2}\right)∼a_{1}\;\frac{D_{Max}}{2}+a_{2}\;\frac{D_{Max}}{2} \end{array}$$

**Figure 3:**
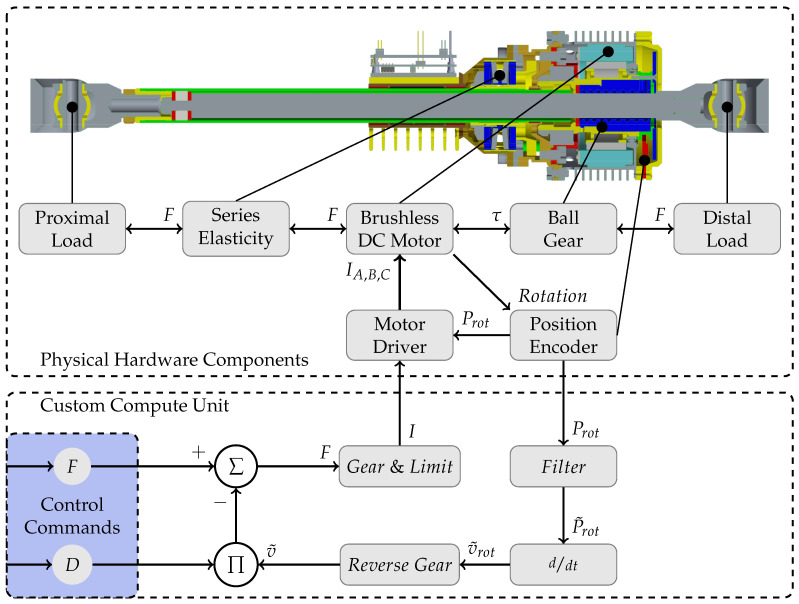
Sketch of the muscular control of an electrical series elastic actuator. On the top, the mechanical construction of the 2nd generation RRLab-Sea is pictured. Attached below, a block diagram of the introduced muscular control concept is given. The two control inputs, force and damping, are highlighted in the lower left corner.

Single muscle activation is mapped to positive or negative force generation $F(a_{1},a_{2})$. Simultaneous activation of both antagonists, i.e., co-contraction, is mapped to a generated damping characteristic $D(a_{1},a_{2})$. With this simple control principle, the required control logic (including feedback loops) of an electrical Sea drastically reduces compared to a complete impedance controller, e.g., [31,50]. Figure 3 sketches the muscular control scheme from digital input commands to the physical current output of the example from 2nd generation RRLab-Sea hardware.

## 3. Distributed Damping Control

In the previous section, muscular control of a series elastic actuator was introduced. In this section, an appropriate control and coordination strategy is developed. The proposed Sea control offers force and damping generation (independent of each other). Hence, an overall actuator coordination strategy can also be split into individual force and damping distribution strategies. In the context of redundant actuation, force distribution strategies were discussed in detail by Nejadfard et al. [41,51,52]. Damping distribution, on the other hand, is mentioned in a quite primitive manner within the original conference publication underlying the article at hand [1]. Hence, in the following, this section provides a detailed analysis of the individual contribution of local muscular damping in the (Cartesian) workspace. Afterward, a new recruitment strategy is introduced to distribute the desired workspace damping among available muscles.

### 3.1. Workspace, Configuration Space, and Actuation Space

**Figure 4:**
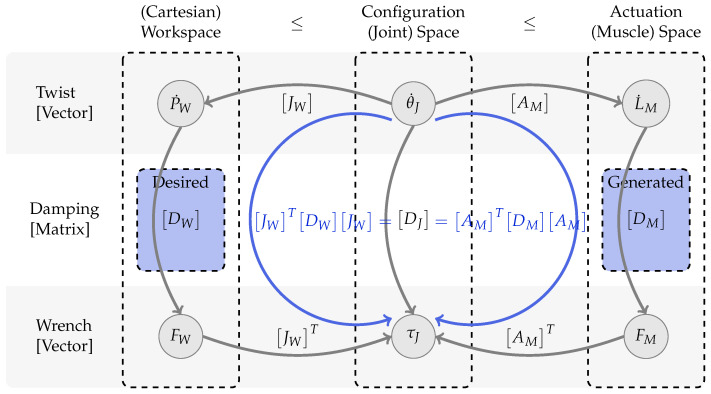
Kinematic coupling of the *(Cartesian) workspace*, the *configuration (joint) space*, and the *actuation (muscle) space* of an arbitrary, robotic limb. The two matrices, $\left[J_{W}\right]$ and $\left[A_{M}\right]$, represent the *position Jacobian* and the *actuator gear* matrices, respectively. All three matrices in the middle $\left[D_{W/J/M}\right]$ represent the appropriately dimensioned, symmetric, positive semidefinite damping matrices. In blue, the desired and generated damping representations are highlighted.

In order to control a robot in an easy and intuitive manner, commands might be given by a desired TCP (*Tool Center Point*, i.e., an arbitrary, maybe virtual, point at the end of the robot’s kinematic chain. In case of controlling a lower limb, this could be, for example, the ankle joint, the heel position, or the tip of the toes) position and motion in the Cartesian workspace representation of the real world. The exact robot motion, however, is executed in the so-called *configuration space*, representing all individual DoFs that are the joints of the robot. As it is not always the case that every joint is mapped to one single actuator, the desired joint motion has to be mapped further to the available actuators, the so-called *actuation space*. Unfortunately, the dimensionalities of all three spaces might increase in the described order, i.e., several motors might operate on the same joint(s) and several joints might operate in the same workspace dimension(s). For a better overview, Figure 4 sketches the kinematic coupling of twist and wrench vectors between these three spaces, together with the respective embedded damping.

From left to right, the abstract but intuitive (Cartesian) workspace, the configuration space, and the actuation space are shown. The workspace on the left is the most simple space that offers an intuitive API for abstract, higher-level limb control. In the middle, the configuration space represents the physical limb configuration by all its joints. On the right, the actuation space contains all actuators available at a limb. Note that the dimensionalities of these three spaces might increase from left to right. Less-or-equal symbols between their headlines indicate dimensional inequalities. Six description vectors, two per space, are visualized as labeled circles. The three in the top row represent combined linear and rotational velocity descriptions (twist vectors), while the ones on the bottom row represent combined force and torque descriptions (wrench vectors). In between these six state vectors, transformation matrices describe their relations.

The two matrices that describe the coupling between the spaces are the *Jacobian* matrix $\left[\begin{array}{c} J_{W} \end{array}\right]$ and the *gear* matrix $\left[\begin{array}{c} A_{M} \end{array}\right]$. These matrices do not need to be invertible due to the different dimensionalities of the spaces. In general, they do not even need to be quadratic. When using linear actuators as motor units, the gear matrix contains the lever arms between the joints and the actuators. The lever arms of the two actuators mounted on the shank of Carl are sketched in Figure 1b. Further details about the lever arms of all actuators on Carl can be found in [52].

The relationship between twist and wrench vectors in all three spaces can be described with a symmetric, positive semidefinite matrix representing non-negative damping. In Figure 4, these damping matrices are shown in the middle row by $\left[\begin{array}{c} D_{W} \end{array}\right]$, $\left[\begin{array}{c} D_{J} \end{array}\right]$ and $\left[\begin{array}{c} D_{M} \end{array}\right]$. The actuation space damping matrix $\left[\begin{array}{c} D_{M} \end{array}\right]$ is a diagonal matrix containing the individual, non-negative actuator damping values ${\vec{d}}_{M}$ on its diagonal: $\left[\begin{array}{c} D_{M} \end{array}\right]=diag\left({\vec{d}}_{M}\right)$. These damping values are passed to the muscular Sea control, introduced in Section 2.2 and visualized in Figure 3. Highlighted in blue, the relations between all three damping matrices are shown in the following equation:

(3) $$J_{W}^{T}\;D_{W}\;J_{W}=D_{J}=A_{M}^{T}\;D_{M}\;A_{M}$$

Since none of the matrices within this equation and due to the above-mentioned properties of the damping matrices, this equation, in general, is not solvable for $D_{M}$, given an arbitrary, desired $D_{W}$ or $D_{J}$ that meets the above requirements. Furthermore, the ’generatable’ damping values per actuator are constrained by physical and electrical limits that must not be exceeded. Hence, an approximation strategy has to be applied to distribute the desired damping to the available actuators.

A simple approach of optimizing within the configuration space has already been introduced in the preliminary work [1] and will be presented in a more detailed and slightly advanced version later in Section 3.3. However, the second potential approach to optimize the actuator damping distribution within the workspace instead of the configuration space has not been explored in previous studies. A brief discussion of this approach and an explanation of why it is generally not feasible are presented below.

### 3.2. Discussion on Individual Muscular Damping in the Cartesian Workspace

To better highlight the upcoming problems when dealing with individual actuators in the workspace, the above equation of damping matrices (3) can strongly be simplified.

Due to the fact that the actuator space damping matrix $D_{M}$ is diagonal, the right-hand side of Equation (3) can be reformulated as a sum of sub-multiples of each actuator’s individual maximum damping:

(4) $$A_{M}^{T}\;diag\left({\vec{d}}_{M}\right)\;A_{M}=\sum_{m}\left(row_{m}{\left(A_{M}\right)}^{T}\cdot row_{m}\left(A_{M}\right)\;{}^{Max}d_{m}\;s_{m}\right)$$

with the function $row_{m}\left(\cdot \right)$ returning the *m*-th row of its matrix argument $\left[\begin{array}{c} \cdot \end{array}\right]$ as a row vector and ${}^{Max}d_{m}\;s_{m}$ representing the *m*-th element of the actuators damping vector ${\vec{d}}_{M}$. ${}^{Max}d_{m}$ is the constant, maximum possible damping of the *m*-th actuator and $s_{m}\in \left[0,1\right]$ represents some fractional part of it.

Similarly, the left side of Equation (3) might be split into an equivalent sum of elements representing each actuator’s contribution in the workspace.

(5) $$J_{W}^{T}\;D_{W}\;J_{W}=\sum_{m}\left(J_{W}^{T}\;{}^{Max}D_{m}\;J_{W}\;s_{m}\right)$$

This way, it is possible to observe the matrix equation for every available actuator individually and unscaled:

(6) $$\begin{array}{cccc} \forall m\; & :\;J_{W}^{T}\;{}^{Max}D_{m}\;J_{W}\;s_{m} & \; & \overset{!}{=}\;row_{m}{\left(A_{M}\right)}^{T}\cdot row_{m}\left(A_{M}\right)\;{}^{Max}d_{m}\;s_{m}\;\\ \Rightarrow \forall m\; & :\;J_{W}^{T}\;{}^{Max}D_{m}\;J_{W} & \; & \overset{!}{=}\;row_{m}{\left(A_{M}\right)}^{T}\cdot row_{m}\left(A_{M}\right)\;{}^{Max}d_{m} \end{array}$$

Moreover, every actuator on its own is a one-dimensional object. Independent of how it is integrated and the chosen level of abstraction, the resulting action has to be one-dimensional. Hence, actuator-specific damping matrices in all three spaces only need to have a single eigenvector with a non-zero eigenvalue. On the right-hand side of Equation (6), this property is given by construction. On the left-hand side, this property requires a simplified but more restrictive representation of the maximum damping matrix in the workspace ${}^{Max}D_{m}:={\vec{e}}_{m}\cdot {\vec{e}}_{m}^{T}$. Using these representations, the matrices on both sides of Equation (6) are characterized in total by their single, non-zero eigenvectors. Therefore, the matrix equation can be simplified further to the following vector equation:

(7) $$J_{W}^{T}\;{\vec{e}}_{m}\overset{!}{=}row_{m}{\left(A_{M}\right)}^{T}\;\sqrt{{}^{Max}d_{m}}$$

Now, it is easy to see that, as stated initially, the existence and uniqueness of a solution on ${\vec{e}}_{m}$ in total depends on the Jacobian matrix $J_{W}$.

**Figure 5:**
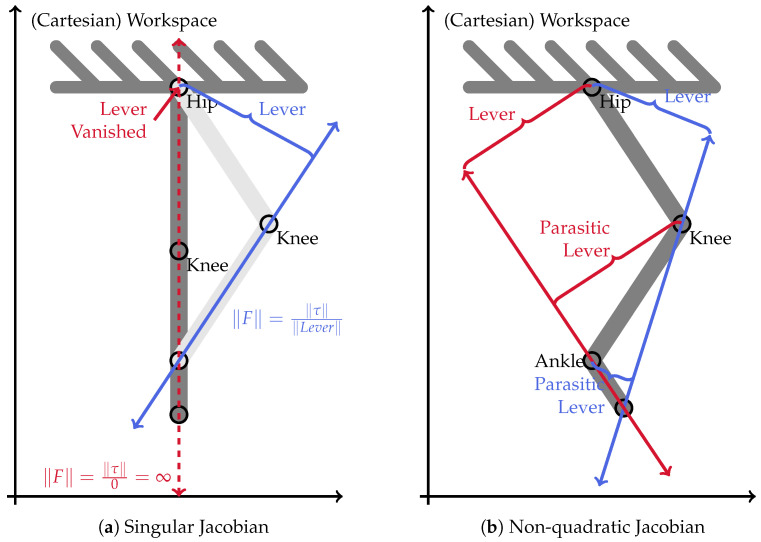
Visualization of upcoming problems of actuator-individual damping in the workspace. Figure (**a**) highlights the problem of singularity in the example of a planar two-link robot. The problem of having a higher-dimensional configuration space than the Cartesian workspace (i.e., a non-square Jacobian) is sketched in figure (**b**) with the example of a planar, three-link robot.

The first case of no solution is the well-known problem of singular positions. Whenever the Jacobian matrix $J_{W}^{T}$ becomes singular, solving Equation (7) results in no and/or infinite solutions. Figure 5a visualizes the problem of singularity at the example of the hip joint actuator of a planar, two-link robot without TCP orientation. As worked out in detail by Nejadfard et al. in 2018 [52], the direction of work of a hip-propelling actuator is parallel to the shank segment of the robot. In Figure 5a, these directions of work are pictured by the blue and the dashed arrows, and the red bidirectional force arrows represent the two sketched robot configurations. This alignment results from the requirement that these forces have to exactly pass through all joints that should not be affected by the hip, i.e., all others. The distance between these vectors and the hip joint embodies the effective lever arm coupling the joint torque and the force in the workspace. As highlighted in the sketch, this lever arm vanishes in the singular position, leading to a theoretically infinite resulting force vector in the workspace. Hence, in a singular position, the effective direction of actuation in the workspace is undefined and cannot be used for further processing.

The second reason that an actuator-individual workspace solution does not exist is when the Jacobian matrix is non-square. This occurs when the configuration space of a robot has a higher dimensionality than the workspace. To visualize this problem, Figure 5b extends the planar, two-link robot of the previous Figure 5b by an ankle joint with a foot segment. The orientation of the new TCP is still not considered. Now, there are two main possibilities to orient the direction of work of the hip in the workspace. Either it can pass through the knee joint as in the two-link robot, or it can pass through the appended ankle. Figure 5b sketches both these variants in blue and red, respectively. It is easy to see that both variants, when applied, cause parasitic torques on the joints that they do not pass through. Hence, to really achieve such a vector of operation in the workspace, the parasitic joint has to be activated, too. Unfortunately, this additional joint itself comes with the exact same problem for its own direction of work which, in general, does not even match the desired one of the original joint.

Hence, the above discussion can be concluded with the following statement. In general, individual actuator (muscle) recruitment does not have proper or unique representation in the workspace for arbitrary robot configurations. Obviously, this statement does not hold for some special cases, e.g., in the case of a *half-singular* Jacobian. Consequently, actuator damping distribution strategies can only be applied in the workspace when the respective Jacobian matrix is guaranteed to be invertible. If this guarantee cannot be given by the robot construction, an alternative strategy has to be applied in the configuration space. Such a suitable approximation strategy is introduced in the following Section 3.3.

### 3.3. Approximation of Muscular Damping in the Configuration Space

Since damping is represented as a quadratic, symmetric, positive semidefinite matrix, the theoretical degree of freedom of a damping matrix is the number of its upper or lower triangular elements. The requirement to only allow non-negative eigenvalues only limits the space of valid solutions within the entire space of available DoF. Hence, to exactly represent a configuration space damping in the actuation space 1/2d(d+1), many independent actuators are required, with *d* being the dimensionality of the configuration space. For the very simple, planar, three-joint robot within Figure 5b, this would already require 6 individual actuators. It can be stated that, in general, every real robot will be underactuated with respect to actuation space damping. An optimization approach to find the best possible actuator damping commands is required. One solution is to find a suitable damping matrix $D_{S}$ within the configuration space that minimizes the weighted error against the desired damping matrix $D_{J}$ in all possible directions.

(8) $$\forall {\dot{\theta }}_{J}\in \{∥{\dot{\theta }}_{J}∥=1\}\;\text{:}\;D_{S}\;{\dot{\theta }}_{J}\overset{!}{=}D_{J}\;{\dot{\theta }}_{J}$$

This can be formulated as a quadratic minimization of the integrated, weighted, squared error. Define:

$$\begin{array}{cc} tr\left(\cdot \right) & \;\mathrm{being}\;\mathrm{the}\;\mathrm{trace}\;\mathrm{of}\;\mathrm{matrix}\;\left[\begin{array}{c} \cdot \end{array}\right]\\ row_{i}\left(\cdot \right) & \;\mathrm{being}\;\mathrm{the}\;i-\mathrm{th}\;\mathrm{row}\;\mathrm{of}\;\mathrm{matrix}\;\left[\begin{array}{c} \cdot \end{array}\right]\\ row\left(\cdot \right) & :=\left(\begin{array}{c} row_{0}\left(\cdot \right),\cdots ,row_{i}\left(\cdot \right) \end{array}\right)\;\mathrm{being}\;\mathrm{the}\;\mathrm{concatenation}\;\mathrm{of}\;\mathrm{all}\;\mathrm{rows} \end{array}$$

The minimization can now be derived as follows:

(9) $$\begin{array}{cccc} \; & \; & \min_{D_{S}}\; & \int_{∥{\dot{\theta }}_{J}∥=1}{∥W\left(D_{S}-D_{J}\right)\;{\dot{\theta }}_{J}∥}^{2} \end{array}$$

(10) $$\begin{array}{ccccc} \Rightarrow & \; & \; & \min_{D_{S}}\; & tr\left({\left(W\left(D_{S}-D_{J}\right)\right)}^{T}\left(W\left(D_{S}-D_{J}\right)\right)\right) \end{array}$$

(11) $$\begin{array}{ccccc} \Rightarrow & \; & \; & \min_{D_{S}}\; & tr\left({\left(WD_{S}\right)}^{T}\left(WD_{S}\right)\right)-2\;tr\left({\left(WD_{S}\right)}^{T}\left(WD_{J}\right)\right) \end{array}$$

(12) $$\begin{array}{ccccc} \Rightarrow & \; & \; & \min_{D_{S}}\; & \frac{1}{2}row\left(WD_{S}\right)\cdot row{\left(WD_{S}\right)}^{T}-row\left(WD_{S}\right)\cdot row{\left(WD_{J}\right)}^{T} \end{array}$$

With the help of the Equations (3) and (4), the weighted, suitable matrix $D_{S}$ can be expressed as a linear combination of all individual damper actuators.

(13) $$\begin{array}{cc} row\left(WD_{S}\right) & =row\left(W\left(A_{M}^{T}D_{M}A_{M}\right)\right)\\ \; & =row\left(W\;\sum_{m}\left(row_{m}{\left(A_{M}\right)}^{T}\cdot row_{m}\left(A_{M}\right)\;d_{m}\right)\right)\\ \; & =row\left(\sum_{m}\left(W\cdot row_{m}{\left(A_{M}\right)}^{T}\cdot row_{m}\left(A_{M}\right)\;d_{m}\right)\right)\\ \; & =\sum_{m}row\left(row_{m}{\left(A_{M}W^{T}\right)}^{T}\cdot row_{m}\left(A_{M}\right)\right)\;d_{m}\\ \; & ={\left[\begin{array}{c} row\left(row_{1}{\left(A_{M}W^{T}\right)}^{T}\cdot row_{1}\left(A_{M}\right)\right)\\ \vdots \\ row\left(row_{m}{\left(A_{M}W^{T}\right)}^{T}\cdot row_{m}\left(A_{M}\right)\right) \end{array}\right]}_{=:L}\;{\vec{d}}_{M} \end{array}$$

Now, the minimization problem can be reformulated to operate directly on the individual actuator damping.

(14) $$\begin{array}{cc} \min_{{\vec{d}}_{M}}\; & \frac{1}{2}\;{\vec{d}}_{M}^{T}\;L\;L^{T}\;{\vec{d}}_{M}-{\vec{d}}_{M}^{T}\;L\;row{\left(W\;D_{J}\right)}^{T}\\ w.r.t.\; & \vec{0}\leq {\vec{d}}_{M}\leq {}^{Max}{\vec{d}}_{M} \end{array}$$

The Hessian matrix $\left(L\;L^{T}\right)$ is only assured as positive semidefinite. With the side condition on the actuator damping vector ${\vec{d}}_{M}$, it is assured to only allow non-negative damping commands for each individual actuator. In this way, the generated damping matrix in the configuration space $D_{S}$ is, by definition, positive semidefinite.

Since the purpose of this control optimization is to deliver a continuous stream of suitable motor control data, it is a better choice to optimize the control change than the actual control values. This way, the optimization result will mostly stay around zero, offering better numerical stability. Furthermore, by adding a diagonal ϵ-matrix to the hessian, the minimization can be guaranteed to be strictly positive definite. In the case of optimizing the control change, this added diagonal matrix acts as a very small exponential (low-pass) filter on the final control commands. The reformulation of Equation (14) to minimize the control change ${\vec{\Delta }}_{M}$ is straightforward. The control vector ${\vec{d}}_{M}$ is replaced by $\left({}^{t-1}{\vec{d}}_{M}+{\vec{\Delta }}_{M}\right)$, with ${}^{t-1}{\vec{d}}_{M}$ being the previous control vector.

(15) $$\begin{array}{cc} \min_{{\vec{\Delta }}_{M}}\; & \frac{1}{2}{\vec{\Delta }}_{M}^{T}\;\left(L\;L^{T}+diag\left(\epsilon \right)\right)\;{\vec{\Delta }}_{M}+{\vec{\Delta }}_{M}^{T}\;\left(L\;L^{T}\;{}^{t-1}{\vec{d}}_{M}-L\;row{\left(W\;D_{J}\right)}^{T}\right)\\ w.r.t.\; & -{}^{t-1}{\vec{d}}_{M}\leq {\vec{\Delta }}_{M}\leq {}^{Max}{\vec{d}}_{M}-{}^{t-1}{\vec{d}}_{M} \end{array}$$

(16) $$\begin{array}{cc} {}^{t}{\vec{d}}_{M} & ={}^{t-1}{\vec{d}}_{M}+{\vec{\Delta }}_{M} \end{array}$$

**Figure 6:**
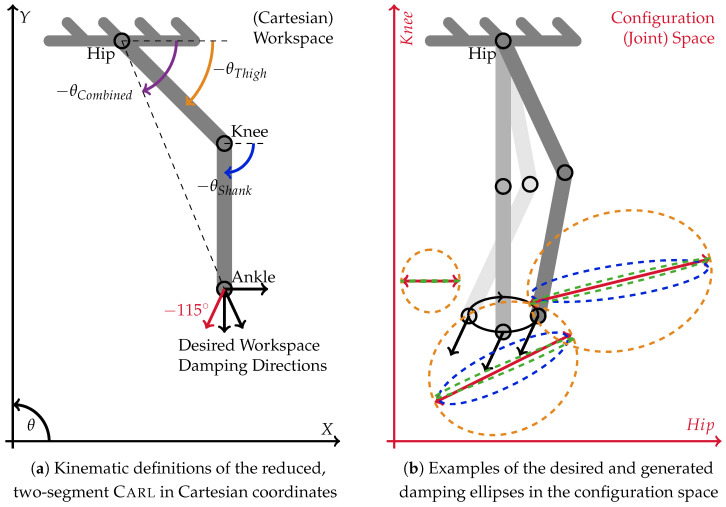
Theoretical experiment setup and result examples of the reduced, two-segment Carl robot. Figure (**a**) pictures the kinematic definitions of the robot in Cartesian coordinates. At the ankle, the four desired damping directions that are approximated individually throughout the experimental ellipsoidal motion are shown. The motion itself is visualized in the background in Figure (**b**). In the foreground, Figure (**b**) shows the desired (solid red double-sided arrows) and generated (dashed, colored) damping ellipses in the configuration space (hip/knee) at three different robot configurations. The desired damping is calculated from the Cartesian damping direction of $-115^{\circ }$ that is highlighted in red within the Cartesian definitions. The blue and orange ellipses are related to the optimizations without weighting and with weighting by the desired damping itself, respectively. In green, the result ellipse is shown, which emerges from the optimized, biarticular lever arm ratio without any further weighting. Muscular damping results over the whole elliptical motion for all three optimization configurations are plotted in Figure 7, Figure 8 and Figure 9.

**Figure 7:**
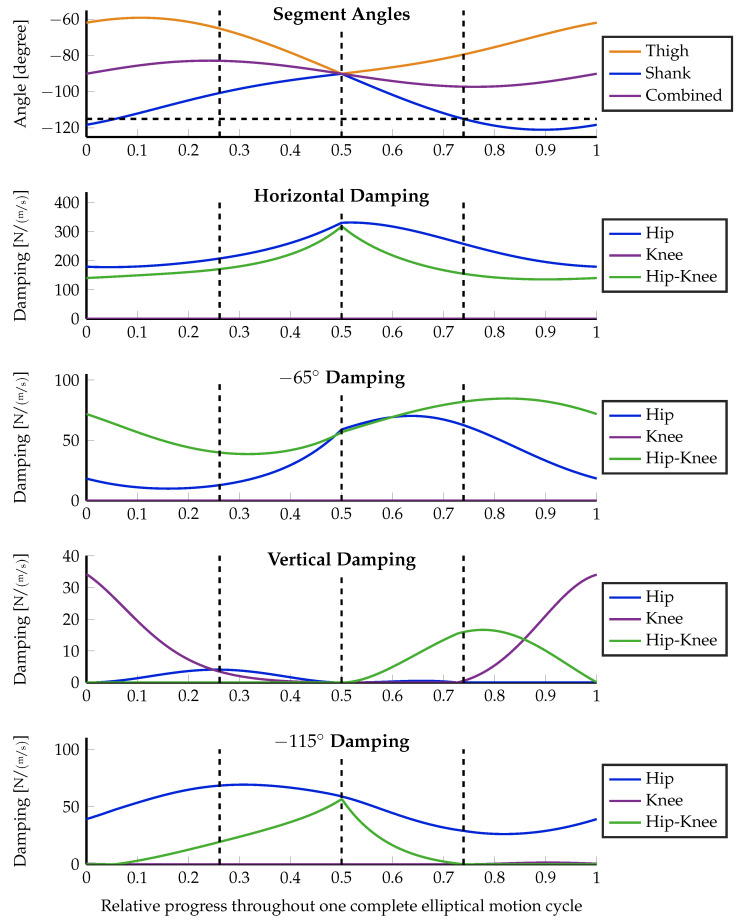
Muscular damping results for all four Cartesian damping directions optimized without special weighting: W=1. On top, the angles of the leg segments as defined in Figure 6a are plotted together with a dashed, horizontal line highlighting the $-115^{{}^{\circ}}$ of the last desired damping direction. The three positions that are visualized in Figure 6b are marked with dashed, vertical lines throughout all five plots. From left to right, these are the front, singular, and back positions. The lower four plots show the damping recruitment of all three actuators independently for the four independent directions of the desired workspace dampings, sketched in Figure 6a. The two-dimensional configuration space representation of the three marked positions for the $-115^{{}^{\circ}}$ Cartesian damping directions are visualized in Figure 6b by the dashed blue ellipses.

**Figure 8:**
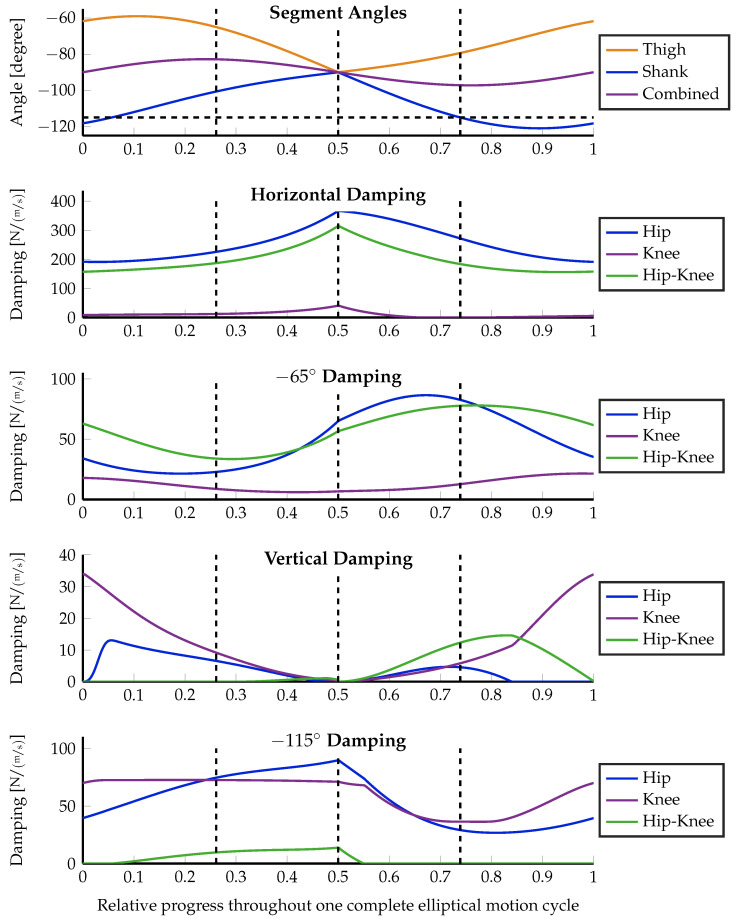
Muscular damping results for all four Cartesian damping directions taking the desired damping vector ${\vec{d}}_{J}$ itself as optimization weight, $W=\left({\vec{d}}_{J}\cdot {\vec{d}}_{J}^{T}\right)\;/\;\left({\vec{d}}_{J}^{T}\cdot {\vec{d}}_{J}\right)$. On top, the angles of the leg segments as defined in Figure 6a and are plotted together with a dashed, horizontal line highlighting the $-115^{{}^{\circ}}$ of the last desired damping direction. The three positions that are visualized in Figure 6b are marked with dashed, vertical lines throughout all five plots. From left to right, these are the front, singular, and back positions. The lower four plots show the result damping recruitment of all three actuators independently for the four independent directions of the desired workspace dampings, sketched in Figure 6a. The two-dimensional configuration space representation of the three marked positions for the $-115^{{}^{\circ}}$ Cartesian damping directions are visualized in Figure 6b by the dashed orange ellipses.

With respect to control stability, it is always safer to over-damp a system than to under-damp it. In order to favor higher damping values over small ones, an interesting choice for the weighting of the optimization is the desired configuration space damping itself $W=D_{J}$. The effect of choosing such a weight is discussed in the following section and illustrated in Figure 6, Figure 7 and Figure 8.

## 4. Discussion on Capabilities and Limits of Actuator-Damped Systems

Three sets of theoretical experiments were executed to evaluate the capabilities and limits of the herein-introduced new control philosophy. The ankle of one leg of the planar, bipedal robot Carl, reduced to its two leg segments, thigh and shank, was moved in an ellipsoidal trajectory. Both segments have identical, natural lengths of 42 cm. The hip and knee joints are limited in their range of motion from each 0° to −120° and −90°, respectively. Detailed descriptions of the construction of Carl can be found in [29,36]. During the ellipsoidal motions, the introduced distribution strategy is applied to approximate four individual, one-dimensional unit-damping vectors in the workspace. The four directions are 0°, −65°, −90°, and −115°. They are visualized as arrows attached to the robot’s TCP in Figure 6a. The damping matrix in the workspace is the outer product of the damping vectors ${\vec{d}}_{W}$ with themselves. Equation (3) is used to translate the workspace damping matrix $D_{W}$ into the configuration space damping matrix $D_{J}$ that is required by the damping distribution algorithm.

(17) $$\begin{array}{cc} D_{J} & =J_{W}^{T}\;D_{W}\;J_{W}\\ \; & =J_{W}^{T}\;{\vec{d}}_{W}\cdot {\vec{d}}_{W}^{T}\;J_{W} \end{array}$$

(18) $$\begin{array}{cc} \; & ={\vec{d}}_{J}\cdot {\vec{d}}_{J}^{T}\\ \mathrm{with}\text{:}\;{\vec{d}}_{J}: & =J_{W}^{T}\;{\vec{d}}_{W} \end{array}$$

By reducing Carl to a two-link configuration, we can design the simplest possible robot that can feature both mono- and multiarticular actuators. This reduction is necessary to limit the resulting data amount and complexity of the algorithm. Accordingly, the four directions of workspace damping are specifically chosen to highlight certain properties of the applied damping distribution algorithm. The horizontal and vertical damping directions represent the primary axes required for upright (bipedal) balance and gravity resistance. Moreover, the horizontal damping direction emphasizes the advantages of properly chosen biarticular actuation. The vertical damping direction also underscores the algorithm’s resistance against singularity, which often causes numerical instabilities. The damping generation at the angle of −115° is chosen to show the effect of being aligned with the shank. In the case of this two-segment robot, this alignment represents the principle direction of work of a monoarticular actuator acting on the hip. The problem of singularity and the principle direction of work are discussed in detail in Section 3.2 and sketched in Figure 5a. The damping direction of −65° highlights the big differences between equally forward and backward (−115°)-pointing desired workspace damping arising from the one-directional knee joint. All results are explained in detail in the following.

**Figure 9:**
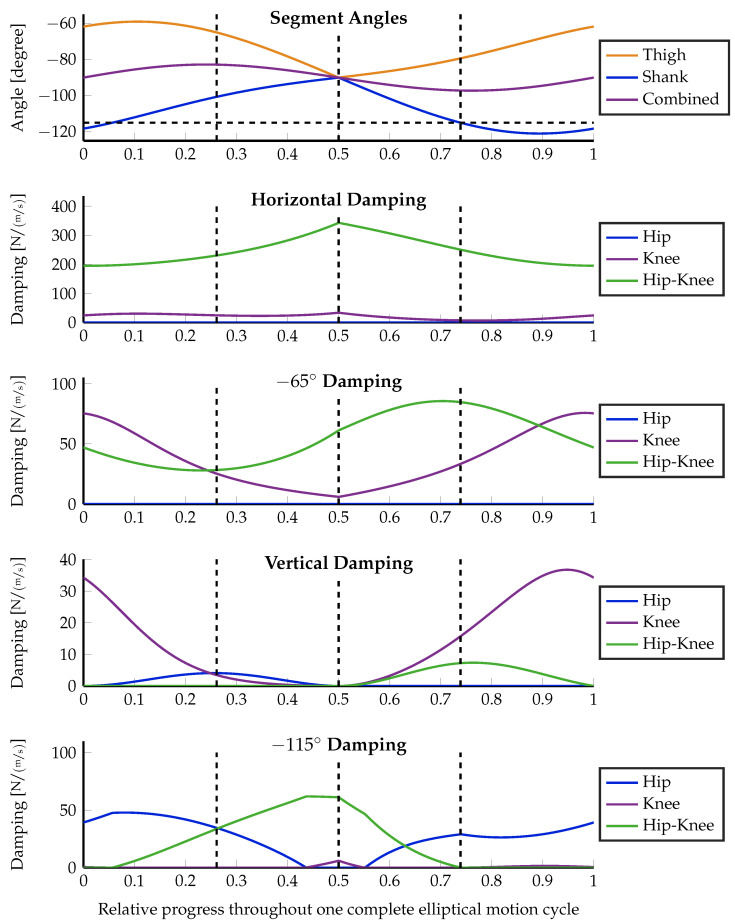
Muscular damping results for all four Cartesian damping directions without special weighting, but with the lever arm ratio of the biarticular hip–knee actuator forced to be 2-to-1 (hip-to-knee) as proposed by Nejadfard et al. [52]. On top, the angles of the leg segments as defined in Figure 6a are plotted together with a dashed, horizontal line highlighting the $-115^{{}^{\circ}}$ of the last desired damping direction. The three positions that are visualized in Figure 6b are marked with dashed, vertical lines throughout all five plots. From left to right, these are the front, singular, and back positions. The lower four plots show the damping recruitment of all three actuators independently for the four independent directions of the desired workspace dampings, sketched in Figure 6a. The two-dimensional configuration space representation of the three marked positions for the $-115^{{}^{\circ}}$ Cartesian damping directions are visualized in Figure 6b by the dashed green ellipses.

The motion starts at the topmost position, moving forward first. In the motion path sketch within Figure 6b, this is a clockwise execution. After half of the motion, the robot passes its singular position with a backward motion direction. In order to keep the influence of the changing lever arm low, the motion is kept small with a horizontal diagonal of 20 cm and a vertical height of 10 cm. The experiment is executed using three different configurations, which highlight important properties of the distributed damping coordination, given in Equation (15). First, the optimization is executed without using an optimization weight matrix: W=1. Second, the desired damping vector in the configuration space ${\vec{d}}_{J}$ itself is taken as additional weight, i.e., $W=({\vec{d}}_{J}\cdot {\vec{d}}_{J}^{T})\;/\;\left({\vec{d}}_{J}^{T}\cdot {\vec{d}}_{J}\right)$. Moreover, the optimization is again executed without weighting, but the lever arm ratio of the biarticular hip–knee actuator is forced to always be 2-to-1 (hip-to-knee) as proposed by Nejadfard et al. in [52]. Figure 6 shows the experimental setup, the definitions, the execution, and the optimization results in the configuration space. Three different optimization results at three concrete Cartesian positions were plotted for the desired Cartesian damping direction of $-115^{\circ }$. Figure 7, Figure 8 and Figure 9 plot all optimized actuator damping values for all four desired Cartesian directions, respective to the three above-mentioned optimization configurations.

As discussed in Section 3.2, and sketched in Figure 5a, the direction of work of the hip joint always aligns with the shank of the two-segment robot. This phenomenon is clearly visible in Figure 6b in the back position. The desired joint damping is a horizontal line. Hence, in this situation, the hip is the only required actuator to perfectly generate the desired Cartesian damping. In the two other positions, there is a clear vertical (knee) component present. Note that the singular position in the middle of the motion does not cause any numerical artifacts or instabilities. This can also be observed in all of the detailed result plots.

Except for the specially constructed back position, the proposed damping distribution strategy in general is not able to exactly match the desired damping. (Note that the blue ellipse of the back position is covered by the green one and, hence, is not visible in the figure.) It can be seen that both the blue and the green ellipses are optimized to be close to the desired one. The errors are ellipses with slightly different radii and orientations. This inability is due to the fact that the generated damping cannot be different from a non-negative, linear combination of the actuator-individual dampings as explained in Equation (4). By definition, the direction of work of monoarticular actuators in the configuration space is fixed. Therefore, the lever arm ratio of biarticular (or multiarticular) actuators is the only possibility to tune the capability of a robot to render the concrete damping at the actuator level. Note that this is a *mechanical* design decision. It is clearly visible that the green ellipses, generated using the improved biarticular lever arm ratio of 2-to-1 for the biarticular hip–knee actuator, are much closer to the desired damping than the blue ones that are using the original, existing levers of Carl. Details about the exact lever arms of Carl can be found in [29,52].

Comparing the weighted orange damping ellipses against the others, it is not difficult to see that weighting highly influences the optimization results. In the case of the unweighted experimental optimization runs, the algorithm balances both the length and the width of the resulting ellipse against the desired one. Consequently, the emerging damping is a little shorter but thicker than the desired one. Against these observations, weighting the optimization with the damping itself results in an optimization that only is interested in matching this single length of the desired damping vector. Everything else is ignored. This effect is clearly visible in Figure 6b. The orange ellipses in all three positions perfectly match the tips of the desired, double-sided vectors shown in red. The desired thickness (zero) is not taken into account at all. Hence, the width of these orange ellipses emerges from the mostly unchanged previous one. However, having one dimension weighted to zero is an extreme scenario that most likely will not occur in real motion scenarios.

From the technical description of Carl in [29], the moment arm ratio of the biarticular hip–knee actuator can be estimated notably below 1 throughout the whole elliptical motion. The detailed analysis of this ratio by Nejadfard et al. [52] gives insight that the direction of work of a biarticular actuator at the hip–knee position with such a small lever arm ratio is quite close to the knee’s one. Consequently, when looking through the result plots in Figure 7 and Figure 8 using the original lever arms of Carl, the knee actuator is mostly unused. Except for the special case of the vertical desired damping, the biarticular hip–knee actuator takes over all responsibilities of the knee. A second exception is the desired damping at $-115^{{}^{\circ}}$ with damping-weighted optimization (see Figure 8). In this case, it is just the other way around. The monoarticular knee is recruited, while the biarticular hip–knee actuator is more or less unused. However, this can be treated as a side effect of the massively deformed generated damping ellipse due to the zero-weighting, as described above.

Comparing both unweighted experiments (Figure 7 and Figure 9), the effect of the changed lever arm ratio of the hip–knee actuator can be observed. The most obvious effect takes place at horizontal damping. Since the lever arm ratio of 2-to-1 works orthogonally to the hip–ankle axis (the herein-named *combined* angle) [52], the horizontal damping is most solely taken over by the hip–knee actuator. Inspecting the muscle recruitment for the slightly forward pointing damping (at $-65^{{}^{\circ}}$), the updated lever arm ratio leads to a recruitment of the knee actuator instead of the hip one, used with the original lever arms. Keeping in mind that the knee actuator acts at the so-called *combined* angle while the hip actuator works at the angle of the shank, it is obvious that recruiting the knee is a much better choice than using the hip actuator for approximating $-65^{{}^{\circ}}$ damping. Unfortunately, in the first experiment (Figure 7), the knee is quite useless due to the working direction of the hip–knee actuator. The only leftover option for the optimization is to use the hip in order to push the resulting damping ellipse toward the desired direction, at the cost of a much higher deviation from the desired. In the case of pure vertical damping, there is no relevant change at all. The monoarticular knee actuator remains the optimal choice for that direction. The same holds for the slightly backward-pointing damping. Whenever the shank is aligned with the desired damping at $-115^{{}^{\circ}}$, the hip actuator takes over that generation solely. The additional knee recruitment in Figure 8 is due to the zero weighting that does not care about any side effects.

At this point, it is not difficult to formulate two main results from these observations:

- The capabilities and limits of a distributed damping strategy at the actuator level are only determined by the mechanical design of the actuator mounts within a robot.
- The higher the variability in the set of available actuators, the higher the possible precision of an approximated distributed damping.

These findings correlate with Schumacher et al.’s conclusion in their review article, i.e., *biarticular muscles in light of template models, experiments and robotics: a review*, from 2020:

“*Thus, the muscle’s mechanical function is strongly influenced by the leg architecture. This coupling also affects the neural coordination of the muscles.*”

[53]

Note that the latter observation result also covers the number of available actuators. Hence, the herein-introduced concept of the distributed damping control at the actuator level offers a novel sense of the existence of muscular variety and the massive over-actuation, present in nature. In addition to the known energy reduction opportunity on torque generation, multiarticular muscles offer the possibility to better generate concrete, desired damping. In this way, unnecessary overdamping in irrelevant directions can be avoided, contributing to less energy-consuming control.

## 5. Actuator-Damping Applied on the Planar Robotic Leg Carl

With controlling damping, the precision of a desired motion (velocity or twist) can be controlled. This way, the damping acts as the proportional gain of a velocity P-controller. However, due to the over-actuation of Carl, it is better to reformulate the controller to use a desired force instead of a desired velocity along with the damping.

(19) $$\vec{F}=D_{0}\;\left({\vec{V}}_{0}-\vec{V}\right)=D_{0}\;{\vec{V}}_{0}-D_{0}\;\vec{V}=:{\vec{F}}_{0}-D_{0}\;\vec{V}$$

The main reasons for going with a desired force are as follows. As shown in Figure 4, the gear matrix $A_{M}$ couples the configuration space and the actuation space. On the velocity side, the calculation of all actuator velocities, given the desired joint velocities, is straightforward. The coupling between joint and actuator motion is mechanically defined, so increasing the dimensionality of the configuration space with respect to the actuation space does not generate additional information. However, when it comes to forces, the coupling goes in the opposite direction, so $A_{M}$ describes a dimensional reduction that may result in information loss. This loss presents the opportunity to choose from various sets of actuator forces without affecting the torques in the configuration space. Different force distribution strategies can be applied to optimize the control effort, energy loss, or other factors. Additionally, passive actuators (e.g., springs) can easily be integrated as additional sources of force or torque. Having passive springs in parallel to active actuators can be used to shift an actuator’s working point out of the zero force. In nature, this effect is achieved by having antagonistic muscles of different sizes and strengths. Two examples of possible distribution strategies were introduced and discussed by Nejadfard et al. in [51].

Since the generation of damping is only an approximation, proper actuator forces ${\vec{F}}_{0}$ can only be calculated once the actually applied damper actuators are known. Therefore, within the configuration space, the desired control, again, has to be the velocity instead of the force. Given the optimized, diagonal actuator damping matrix $D_{M}$ and some arbitrary, desired joint velocities vector ${\dot{\vec{\theta }}}_{J}$, appropriate actuator forces are calculated by solving the following system of equations for ${\vec{F}}_{M}$:

(20) $$A_{M}^{T}\;D_{M}\;A_{M}\;{\dot{\vec{\theta }}}_{J}={\vec{\tau }}_{J}=A_{M}^{T}\;{\vec{F}}_{M}$$

**Figure 10:**
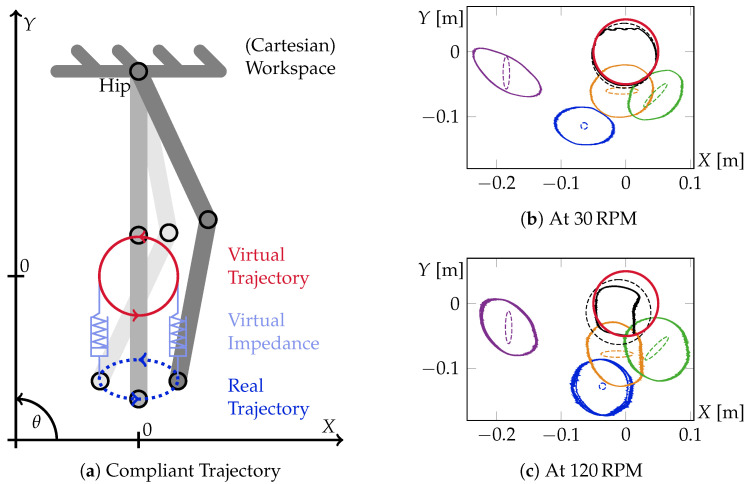
Sketch and experimental results of the so-called compliant trajectory control applied to the ankle of the planar, compliant robotic leg Carl as first presented in [1]. On the left, figure (**a**) visualizes the working principle of the compliant trajectory control. In red, the commanded circular reference trajectory is shown. The virtual spring–damper element and a potential real resulting ankle trajectory are sketched in blue underneath. On the right-hand side, Figures (**b**,**c**) plot the captured, real ankle trajectories of Carl in workspace coordinates at different virtual impedances at speeds of 30 RPM and 120 RPM respectively. Centered at the origin, the shared circular trajectory is given in red. The two-dimensional damping matrices of each of the five individual experimental runs per speed are plotted as dashed ellipses of the same color as the appropriate resulting ankle trajectories. The five damping matrices are: $\mathrm{Blue}\text{:}\left[\begin{array}{cc} 50 & 0\\ 0 & 50 \end{array}\right]$, $\mathrm{Purple}\text{:}\left[\begin{array}{cc} 50 & 0\\ 0 & 250 \end{array}\right]$, $\mathrm{Orange}\text{:}\left[\begin{array}{cc} 250 & 0\\ 0 & 50 \end{array}\right]$, $\mathrm{Green}\text{:}\left[\begin{array}{cc} 150 & 100\\ 100 & 150 \end{array}\right]$, and $\mathrm{Black}\text{:}\left[\begin{array}{cc} 500 & 0\\ 0 & 500 \end{array}\right]$.

Using damping and force, a very simple, so-called *compliant trajectory* control can be set up, i.e., the equilibrium position of a virtual spring–damper element is moved along a desired trajectory. The actual object to be moved is attached to that virtual spring–damper element. A sketch showing the principle of such a compliant trajectory, including the virtual spring–damper element, is given in Figure 10a. Due to the usage of velocity instead of force, as explained above, the stiffness of the virtual spring has to be split into a *velocity*-stiffness followed by the damping

(21) $$K:=D_{W}\;K_{v}$$

with $D_{W}$ being the desired Cartesian workspace damping. The control velocity ${\vec{v}}_{W}$ is then calculated from the position delta ${\vec{\Delta }}_{W}$ in the Cartesian workspace using the *velocity*-stiffness matrix $K_{v}$

(22) $${\vec{v}}_{W}=K_{v}\;{\vec{\Delta }}_{W}$$

To keep the control stable, $K_{v}$ should be defined to at least achieve critical damping. Under the simplification of considering the whole system as a simple, damped spring-mass system with mass *m*, $K_{v}$ can be derived as follows:

(23) $$\begin{array}{cc} 2\;\sqrt{K\;m} & =D_{W}\\ \sqrt{D_{W}\;K_{v}\;m} & =\frac{1}{2}D_{W}\\ D_{W}\;K_{v}\;m & =\frac{1}{4}D_{W}^{2} \end{array}$$

(24) $$\begin{array}{cc} K_{v} & =\frac{1}{4m}D_{W} \end{array}$$

Note that this definition requires the exact desired Cartesian damping $D_{W}$. Unfortunately, the damping is known not to be generated precisely by the actuators. Hence, the estimated mass *m* should be a little overestimated in order to keep the stiffness small enough for proper damping values.

To evaluate the functionality of the herein-introduced distributed damping strategy, two sets of differently damped, circular, compliant trajectories have been executed at the ankle of Carl [1]. Kinematic details of Carl are introduced at the beginning of Section 4. A sketch of the principle setup and the experimental outcomes are visualized in Figure 10. In all experiments, a virtual, circular compliant trajectory with a radius of 5 cm was executed about 63 cm straight below the hip at two different speeds, with five different desired damping matrices $D_{W}$. The individual, color-coded damping matrices are: $\mathrm{Blue}\text{:}\left[\begin{array}{cc} 50 & 0\\ 0 & 50 \end{array}\right]$, $\mathrm{Purple}\text{:}\left[\begin{array}{cc} 50 & 0\\ 0 & 250 \end{array}\right]$, $\mathrm{Orange}\text{:}\left[\begin{array}{cc} 250 & 0\\ 0 & 50 \end{array}\right]$, $\mathrm{Green}\text{:}\left[\begin{array}{cc} 150 & 100\\ 100 & 150 \end{array}\right]$, and $\mathrm{Black}\text{:}\left[\begin{array}{cc} 500 & 0\\ 0 & 500 \end{array}\right]$.

All damping matrices are given in the standard damping SI-unit, force per velocity $\left[\frac{N}{m\;/\;s}\right]$.

The respective configuration space damping matrices $D_{J}$ are calculated as described by Equation (17) in Section 4.

The captured, real executed ankle trajectories at all five desired damping matrices are shown for the two different trajectory execution speeds of 30 RPM and 120 RPM in Figure 10b and Figure 10c, respectively. The ankle positions for all experiments were recorded at a frequency of 1 kHz and were plotted without any post-processing. The estimated mass parameter that was used for calculating the virtual stiffness, as described in Equation (23), is commonly set to 15 kg. That is about half the weight of the whole bipedal robot Carl.

All damping matrices are given in standard damping SI-units, i.e., force per velocity $\left[\frac{N}{m\;/\;s}\right]$. Due to gravity, the real ankle trajectory is expected to be below the virtual reference one.

Both figures, Figure 10b,c show the effects of the different impedances on the real ankle trajectories. As expected, due to gravity, the real trajectories are, in general, below the virtual reference one that is pictured as a red circle. Moreover, it can be seen that increasing damping reduces the error in that direction. To do that, the blue trajectory (i.e., the lowest one that refers to the smallest damping ellipse) is taken as a reference trajectory. Independent of the execution speed, the purple, orange, and green experiments visualize the effects of increased impedance in vertical, horizontal, and diagonal directions, respectively.

However, the purple trajectory is of special interest. It is clearly recorded that not only the vertical direction is increased, as desired, but the horizontal error increases at the same time. An explanation of this effect delivers a closer look at the physical leg configuration in that situation. In order to move the ankle of the two-link system upwards, the knee joint has to be bent forward. That, however, leads to the side effect of moving about half of the mass of the leg forward, too. As a direct consequence, the gravity force that acts on the leg’s shifted center of mass induces a backward torque of the whole leg at the hip. Due to the absence of any gravity-compensation mechanism or similar, this change of the physical properties of the leg hence directly influences the real executed trajectory.

The opposite behavior can be observed in the orange experiment. Unlike the purple trajectory, the orange experiment shows an error reduction in the direction where the desired impedance is not increased. This time, a proper explanation for that effect can be found with a closer look at the damping distribution strategy, as discussed in detail in the previous Section 4. By construction, the generation of horizontal damping always comes at the cost of an inherent vertical one. Consequently, the desired reduction of the horizontal error is achieved by the optimization strategy at the cost of additionally reducing the vertical error, although not desired. Note that this effect is only visible due to the absence of gravity compensation.

The last experiment, the black one, encodes a substantial increment of the desired impedance uniformly in both directions. It was chosen high enough to partially push the actuators and the links of Carl to their physical limits. The flat upper part of the black trajectory at 30 RPM occurs due to the knee joint limit of Carl that occurs at $-90^{{}^{\circ}}$. Aside from that, the desired effect of a much more precise motion is achieved clearly. The recorded trajectory is very close to the commanded virtual one.

Aside from gravity, dynamic effects influence the executed real trajectory. Such dynamic effects can be observed by comparing the two plots of different velocities against each other. First of all, it can be observed that the velocity does not have that much of an effect on the general shape and location of the executed trajectory. The main difference is the variety of motion. This effect is best visible in the blue, the most compliant trajectory. It can be seen that the blue plot at 120 RPM comes at a much higher divergence within the repetitive circular motion compared to the one at 30 RPM.

Moreover, the massive deformation in the most precise trajectory execution, the black experiment, is noticeable at a high speed. An explanation for this observation can be given by the robot’s segment configuration. As mentioned earlier, during the execution of the black trajectory, the robot operates at the boundary of having a maximum possible bent knee. Therefore, a lot of mass is widely extended to the front, which is out of the primary leg axis from hip to ankle. Unfortunately, this creates a long lever for undesired dynamics to act on the robot. On having a closer look at the orange and green trajectories in Figure 10c, this effect, although much smaller, can already be observed on both of these trajectories.

Overall, within this paper, the distributed damping control was evaluated to behave as expected. It is shown that it is possible to generate and control compliant motions in a predictable manner.

## 6. Conclusions and Outlook

Previous work on the bio-inspired actuation system of Carl already provides great insight into the individual contribution of force-emitting muscles [51,52]. Every available actuator, including monoarticular and multiarticular actuators, offers a specific direction of work that can be recruited by any arbitrary motor coordination system. Obviously, the more different types of actuators are present, the more opportunities for recruitment exist. Hence, at the cost of control effort, over-actuation can be used to improve a robot’s overall energy efficiency. However, although energy reduction is an important feature, it does not really contribute to a robot’s movement capabilities. Furthermore, the possibility of energy reduction by over-actuation strongly depends on the actuator’s mounting positions, the internal state of the limb configuration, and the requested control. Hence, energy efficiency can be treated as not being the only reason for the existence of the massive over-actuation of natural limbs. The capability of precisely generating distributed impedance at the muscular level requires a variety of available actuators. The larger and more diverse the set of available actuators, the better the desired damping can be approximated. However, when compensating for missing damping capabilities by over-damping, it again breaks down to energy reduction.

Analogous to the analysis of muscle force contributions, the contribution of muscular damping can be analyzed. This way, it has been shown that the possible shape of a muscle-generated, distributed workspace impedance mostly depends on the pose of a limb (and the internal actuator mounting). As described in Section 2, identical observations on human arms were made by Mussa-Ivaldi et al. in 1985 [43]. Following the same argumentation of using over-actuation for force generation, every available actuator adds its own unique capability to the overall control of a limb. Hence, the more muscular variety that is available, the better and more accurate a desired damping can be generated at a muscular level.

The novel muscular control principle introduced within this article is widely backed by reasoning against observed, natural characteristics. As a result, the overall observable behavior of the proposed muscular control strategy comes with similar characteristics as can be observed in nature. With theoretical experiments on the kinematic structure of Carl, the capabilities and the limits of the novel, distributed damping strategy have been highlighted. Experimental results on the real bipedal robot Carl have been executed to prove the novel control strategy to be functional and stable [1]. Observed, undesired motion perturbations were clearly traced back to the results as well as observations of the theoretical experiments. To the best of our knowledge, no other legged robot has been equipped and controlled with such a distributed, muscular damping control approach. Most similar to the concept introduced here is the control approach proposed by Sharbafi et al. in [54]. Within that work, the so-called *BioBiped 3* robot was controlled by altering the mounting positions of springs spanning across the robot’s joints.

One next step to evaluate the general capability of distributed damping is to bring this control approach into more complex motions. Moreover, further improvements on the basic functionality can be made by introducing gravity or dynamics compensation mechanisms.

## Data Availability

Not applicable.
